# Predictive structure building in language comprehension: a large sample study on incremental licensing and parallelism

**DOI:** 10.1007/s10339-023-01130-8

**Published:** 2023-03-16

**Authors:** Hiroki Fujita

**Affiliations:** Department of Linguistics, University of Potsdam, Karl-Liebknecht-Straße 24–25, 14476 Potsdam, Germany

**Keywords:** Prediction, Incremental licensing, Parallelism, Garden-path, Parsing, Language comprehension

## Abstract

In online language comprehension, the parser incrementally builds hierarchical syntactic structures. The predictive nature of this structure-building process has been the subject of extensive debate. A previous study observed that when a wh-phrase indicates parallelism between the upcoming wh-clause and a preceding clause (e.g., *John told some stories, but we couldn’t remember which stories…*), the parser predictively constructs the wh-clause. This observation demonstrates predictive structure building. However, the study also suggests that the parser does not make a prediction when the wh-phrase indicates that parallelism does not hold (e.g., *John told some stories … with which stories…*), a potential limit to the prediction of syntactic structures. Crucially, these findings are controversial because the study did not observe processing difficulty when disambiguating input indicated that the predicted continuation was inconsistent with the globally grammatical structure (*garden-path effects*). The controversial results may be due to a lack of statistical power. Therefore, the present study conducted a large-scale replication study (324 participants and 24 sets of materials). The results revealed that the parser predicts the clausal structure, irrespective of the type of wh-phrase. There was also evidence of garden-path effects, supporting the finding that the parser makes a prediction. These observations suggest that the prediction algorithm inherent in the human parser is more powerful than assumed by the previous study and that the parser attempts to construct globally grammatical structures during revision.

## Introduction

During online language comprehension, the parser analyses each word and incrementally constructs hierarchical syntactic structures. Research has argued and observed that online language comprehension is a predictive process; the comprehender generates hypotheses about incoming elements during sentence processing (e.g., Crocker 1996; Fujita and Cunnings 2022, in press; Gibson 1991; Gorrell 1995; Ito et al. 2016, 2020; Kamide and Mitchell 1999; Kimball 1973; Lau et al. 2006; Omaki et al. 2015; Weinberg 1993). The present study investigates and discusses the mechanism underlying this predictive process focusing on sentence parsing.

There is evidence that structure building is a predictive process (e.g., Aoshima et al. 2004, 2009; Kush et al. 2017; Phillips 2006; Staub and Clifton 2006; Yoshida et al. 2013). Crucial to the present study is Yoshida et al. (2013). Yoshida et al. investigated whether the parser predictively constructs a clausal structure that follows a wh-phrase, as in the substring below.

1. John told some stories, but we couldn’t remember which stories…

In (1), the wh-phrase “which stories” indicates that a clause (TP) follows. This wh-clause must contain a subject noun phrase (NP), a verb phrase (VP) and the wh-phrase’s base position (t), with appropriate lexical content, as in *we couldn’t remember [*_*NPt*_* which stories] [*_*TP*_* [*_*NP*_* you] [*_*VP*_* heard t]]* (Chomsky 1977; Ross 1969). Crucially, in (1), the wh-clause’s entire content is recoverable from the first clause because the two clauses can be parallel in syntactic structure and lexical content (i.e., *John told some stories, but we couldn’t remember which stories John told*). This recoverability does not hold if a preposition accompanies the wh-phrase and undermines the parallelism, as below.^1^

(2) John told some stories, but we couldn’t remember with which stories…

Yoshida et al. (2013) investigated whether the parser predictively constructs the clausal structure and recovers its content from the first clause by utilising *connectivity effects* (Merchant 2005; Stjepanović, 2008; Truswell 2014) and *online reflexive resolution* (Sturt 2003).^2^ Connectivity refers to a phenomenon where a fronted phrase appears to occupy a lower position. Online reflexive resolution is a process where the parser resolves a reflexive by searching for its antecedent during sentence processing. For example, consider the following sentences.

- John/Mary told some stories, but we couldn’t remember
- which stories about himself Tom became impressed with.
- with which stories about himself Tom became impressed.

These sentences are akin to the substrings in (1/2) but have an overt clausal continuation after the wh-phrase. Also, the wh-phrase in (3a/b) contains a reflexive (“himself”), which depends referentially on a *c-commanding* NP in its *binding domain* (*Binding Principle A*; Chomsky 1981). C-command refers to a structural relation between nodes. The present study posits that x c-commands y if and only if x does not dominate y and x’s parent dominates y (Reinhart 1976). The binding domain for x is the minimal NP or TP containing x, x’s governor and a subject, of which x is not a part (Chomsky 1981, 1986). According to these definitions, the reflexive in (3a/b) corefers with the wh-clause subject NP (“Tom”) because this NP c-commands it in its binding domain due to connectivity effects (e.g., [_CP_ [_NPt_ which stories about himself] [_TP_ Tom became impressed with t]]). However, in (3a), if the parser predicts the clausal structure upon encountering the wh-phrase and recovers its content from the first clause, the first clause subject NP must serve as the antecedent (until the wh-clause subject NP appears, e.g., [_TP_ [_NPk_ John/Mary]] … [_CP_ [_NPt_ wh [_NPk_ himself]] [_TP_ [_NPk_] [_VP_ t]]]). In (3a/b), the first clause subject NP either matches (“John”) or mismatches (“Mary”) the reflexive’s gender. It is known that the parser searches for a structurally licensed antecedent immediately after encountering a reflexive and that processing difficulty ensues when the two NPs disagree in gender (*gender mismatch effects*; e.g., Sturt 2003). Research often utilises reading times as an index of processing difficulty, assuming that reading times become longer as processing difficulty increases. Therefore, in (3a), if the parser predicts the wh-clause and recovers its content from the first clause, reading times at the reflexive should be longer in the gender-mismatch (“Mary…himself”) than gender-match (“John…himself”) conditions. In (3b), Yoshida et al. expected gender mismatch effects to be absent because the prepositional wh-phrase undermines parallelism between the two clauses, thereby preventing the recovery of the wh-clause’s entire content from the first clause. In a self-paced reading task, Yoshida et al. confirmed these hypotheses; they observed gender mismatch effects at the reflexive in (3a) but not in (3b).

Yoshida et al.’s (2013) results have several implications for sentence parsing theories. One is that the parser predictively constructs a large amount of syntactic structure. This implication follows from the fact that the predicted representation comprises a clause ([_CP_ wh [_TP_ [_NP_] [_VP_]]]). Another is that the parser preferentially reuses material in the left context. At the wh-phrase in (3a), it is not impermissible to construct the wh-clause without recovering its content from the first clause. Yoshida et al. argue that the parser recycles material because it prefers to maximise parallelism between the clauses ([_TP_ John told some stories] … [_TP_ John told which stories]; see also Carlson 2001; Frazier et al. 1984; Hall and Yoshida 2021; Kim et al. 2020; Knoeferle and Crocker 2009).

Another implication, which is crucial for the present study, is that the parser does not predict the clausal structure when the wh-phrase indicates that parallelism between the two clauses does not hold. As noted earlier, Yoshida et al. (2013) hypothesised that gender mismatch effects would be absent in (3b) because the prepositional wh-phrase prevents the recovery of the wh-clause’s entire content from the first clause. However, this unrecoverability does not disallow the parser to construct the clausal continuation and posit joint reference between the subject NPs of the first clause and the wh-clause at the wh-phrase (i.e., it is possible to analyse (3b) as [_TP_ [_NPk_] [_VP_ V1]] … [_CP_ [_PPt_] [_TP_ [_NPk_] [_VP_ V2 t]]], V1 ≠ V2). Given that the reflexive in (3b) corefers with the wh-clause subject NP, the absence of gender mismatch effects might indicate that the parser does not predictively construct the clausal structure. In other words, Yoshida et al.’s observations might point to a potential limit to the prediction of syntactic structure. If this interpretation holds, we must assume that, in some circumstances, the parser predicts a clausal structure only when parallelism provides a cue for it (i.e., prediction is a parallelism-driven process). This potential limitation is theoretically crucial, given that the clausal structure following the wh-phrase is necessary for the sentence’s well-formedness and that there is evidence and argument that the parser predictively constructs obligatory structures during sentence processing (*incremental licensing*; Abney 1986; Aoshima et al. 2004; Crocker 1996; Frazier and Clifton 1996; Gibson 1991; Gibson et al. 1994; Gorrell 1995; Pritchett 1988, 1991, 1992; Weinberg 1999). That is, according to the incremental licensing theory, the parser should predictively construct the clausal structure upon encountering the wh-phrase in both (3a) and (3b), and Yoshida et al.’s observations in (3b) might contradict this theory.

Alternatively, the absence of gender mismatch effects in (3b) may indicate that the parser predicts the wh-clause but posits disjoint reference with the first clause subject NP (e.g., no prediction of lexical content; [_NPk_ John/Mary] … [_CP_ [_PPt_ wh] [_TP_ [_NPi_] [_VP_ t]]]). This interpretation is compatible with the incremental licensing theory, and if it is valid, we must explain why the parser avoids coreference at the wh-phrase.

As described above, Yoshida et al.’s (2013) results have significant implications for sentence parsing theories. However, there is one concern about their results: in (3a), their participants did not show processing difficulty at the wh-clause subject NP in the gender-match condition. The appearance of this NP indicates that the material recovered from the first clause is incompatible with the globally grammatical structure. Crucially, there is substantial evidence that reading times increase when an input word does not fit into the current structure (e.g., Clifton 1993; Cunnings and Fujita 2021; Frazier and Rayner 1982; Fujita 2021b; Fujita and Cunnings 2020, 2021a, 2021b; Slattery et al. 2013; Sturt et al. 1999; Tabor and Hutchins 2004). This *garden-path effect* (Frazier and Rayner 1982) is assumed to result from the parser’s difficulty integrating disambiguating input into the current structure and its attempt to construct the globally grammatical structure (*revision*). Given these studies, we can expect some processing difficulty at the wh-clause subject NP in (3a). Thus, the absence of garden-path effects at the wh-clause subject NP potentially contradicts the finding that the parser predicts the clausal structure. If gender mismatch effects observed in Yoshida et al. are an experimental artefact, and if their observations at the disambiguating region represent the underlying mechanism of sentence parsing, we must assume that the human parser is not powerful enough to predict such a large amount of syntactic structure as a TP, at least in some circumstances.

There are, however, other accounts of why Yoshida et al. (2013) did not observe garden-path effects. One is that the parser does not initiate the revision process or halts it promptly upon disambiguation because the predicted continuation is tolerable after disambiguation. In psycholinguistics, some take this line of approach. For example, *the Good-Enough approach* views language comprehension as a heuristic rather than an algorithmic process and presupposes that the comprehender employs fast and frugal heuristic procedures even when these procedures do not conform to the principles of grammar (e.g., Christianson et al. 2001; Ferreira and Patson 2007; Slattery et al. 2013). One consequence of this presupposition is that the comprehender creates representations incompatible with globally grammatical structures. Following the Good-Enough approach, we could interpret the absence of garden-path effects in (3a) as indicating that the predicted continuation is good enough to comprehend the sentence. If sentence processing follows simple heuristic procedures, we need to specify under what circumstances the parser omits the revision process and what structure it constructs (e.g., where does disambiguating input attach?).

Alternatively, the contrasting findings may be due to a lack of statistical power. Yoshida et al. (2013) conducted a self-paced reading experiment with 40 participants and 24 sets of materials. These numbers are typical in sentence processing research. However, given that experimental materials tested in Yoshida et al. are structurally complex, precise estimates of the effect may require high statistical power. Therefore, the present study conducted a high-power replication of Yoshida et al. using a lexicality maze task with 324 participants and 24 sets of materials.

## Methods

### Participants

The experiment, conducted online, involved 324 native English speakers recruited via Prolific (https://www.prolific.co). These participants were over 18 years old, grew up and lived in the UK and were British citizens.

### Design and materials

The experiment contained 24 sets of experimental materials from Yoshida et al. (2013), as in (4a–d) below.

- *Wh-NP*, *Gender match.*

Janet’s grandfather told some stories at the family reunion, but we couldn’t remember which stories about himself from the party the brother was so very impressed with.

(4b) *Wh-NP*, *Gender mismatch.*

Justin’s grandmother told some stories at the family reunion, but we couldn’t remember which stories about himself from the party the brother was so very impressed with.

(4c) *Wh-PP*, *Gender match.*

Janet’s grandfather told some stories at the family reunion, but we couldn’t remember with which stories about himself from the party the brother was so very impressed.

(4d) *Wh-PP*, *Gender mismatch.*

Justin’s grandmother told some stories at the family reunion, but we couldn’t remember with which stories about himself from the party the brother was so very impressed.

In (4c/d), a preposition accompanies the wh-phrase, but not in (4a/b). In (4a/b), the wh-clause’s entire content is recoverable from the first clause until the wh-clause subject NP appears. The first clause subject NP matches the reflexive’s gender in (4a/c) and mismatches in (4b/d).

If structure building is a predictive process, three hypotheses are conceivable. One is that the parser predicts the wh-clause only when parallelism provides a cue for the clausal structure. If this hypothesis is correct, gender mismatch effects at the reflexive should only occur in the wh-NP conditions, with longer reading times in (4b) than (4a). Besides, this reading time pattern should reverse in direction at the disambiguating region (“brother”) due to garden-path effects (e.g., Frazier and Rayner 1982). Similar results should be obtained if the parser predicts the clausal structure in both wh-NP and wh-PP conditions but posits joint reference between the subject NPs of the first clause and the wh-clause only in the wh-NP conditions. If the parser predictively constructs the wh-clause and assumes coreference irrespective of the presence or absence of the preposition, gender mismatch effects and garden-path effects should occur in both wh-NP and wh-PP conditions. Thus, what is crucial is whether a significant main effect of gender or a significant wh-type by gender interaction appears at the reflexive and disambiguating regions.

### Procedure

**Fig. 1:**
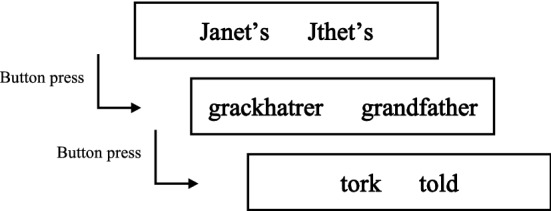
An example of the lexicality maze task

The present study employed a lexicality maze task (Forster et al. 2009; Witzel et al. 2012). In this task, participants read each sentence word by word, with each word presented with a pseudoword, and needed to press a button corresponding to the correct word (see Fig. 1). Thus, the data obtained from the maze task include reading times and judgement reaction times. For expository purposes, the present study refers to this measure as reading times. When participants chose a pseudoword, the trial was immediately terminated, and the next trial began. The lexicality maze task was administered in PCIbex Farm (Zehr and Schwarz 2018), and the experimental file was created using code available online (Boyce et al. 2020; Fujita 2021a). The experiment began with four practice trials, followed by 24 experimental sentences and 72 fillers presented in a pseudorandomised order.

### Data analysis

The dependent variable was log-transformed reading times at four regions. These regions were the reflexive (“himself”), post-reflexive (“from”), disambiguating (“brother”) and post-disambiguating (“was”) regions. Before data analysis, reading times shorter than 300 ms or longer than 7000 ms were excluded.^3^ These outliers represented less than 0.01% of the data. For data analysis, linear mixed-effects models with full variance–covariance matrices for the random effects (*the maximal model*; Barr et al. 2013) were fitted separately for each region using the lme4 package (Bates et al. 2015) in R (R Core Team 2020). The fixed effects were sum-coded (0.5/–0.5) main effects of wh-type (wh-NP/wh-PP), gender (match/mismatch) and their interactions. When the maximal model did not converge, random effect correlations were initially removed, and then, random effects with the least variance were iteratively removed until the model converged. To interpret the results, *p* values were estimated from the *t* distribution (Baayen 2008), and those below 0.05 were interpreted as statistically significant. Data and analysis code are available at https://osf.io/rh4xz.

## Results

**Fig. 2:**
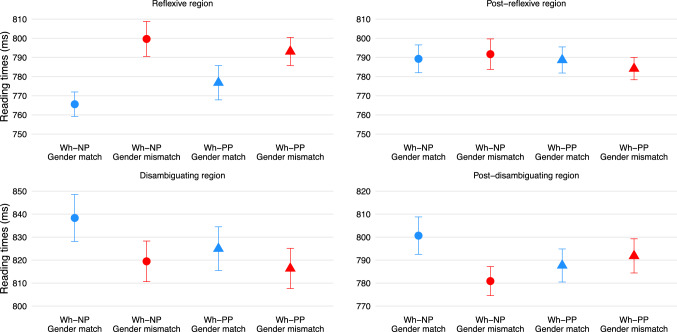
Reading times at the (post-)reflexive and (post-)disambiguating regions. Error bars are standard errors

Table 1 summarises statistical analyses, and Fig. 2 visualises reading times at the (post-)disambiguating and (post-)reflexive regions.

**Table 1:** A summary of statistical analyses at the (post-)reflexive and (post-)disambiguating regions

	Estimate	SE	*t*	*p*	Estimate	SE	*t*	*p*
	Reflexive region				Post-reflexive region			
Intercept	6.606	0.01	567.96	< 0.001	6.622	0.01	778.6	< 0.001
Wh-type	0.001	0.01	0.13	0.898	0.000	0.01	0.04	0.969
Gender	**− 0.029**	**0.01**	**− 4.64**	** < 0.001**	− 0.000	0.01	− 0.05	0.960
Wh-type x Gender	0.001	0.01	0.06	0.955	0.001	0.01	0.07	0.943
	Disambiguating region				Post-disambiguating region			
Intercept	6.650	0.03	206.28	< 0.001	6.624	0.02	330.39	< 0.001
Wh-type	0.009	0.01	1.08	0.282	0.004	0.01	0.60	0.546
Gender	**0.013**	**0.01**	**2.10**	**0.036**	0.006	0.01	1.04	0.297
Wh-type x Gender	0.015	0.01	1.07	0.286	**0.025**	**0.01**	**2.07**	**0.039**

### Reflexive region

Analysis revealed a significant main effect of gender, with longer reading times in the gender-mismatch than gender-match conditions. The wh-type by gender interaction was not statistically significant.

### Post-reflexive region

No effects were statistically significant.

### Disambiguating region

There was a significant main effect of gender, with longer reading times in the gender-match than gender-mismatch conditions (i.e., garden-path effects).

### Post-disambiguating region

Analysis showed a significant wh-type by gender interaction. As a follow-up analysis, an additional model with nested contrasts was fitted to examine the effect of gender within each level of wh-type. This analysis revealed garden-path effects in the wh-NP conditions (Estimate = 0.019, SE = 0.01, *t* = 2.19, *p* = 0.29) but not in the wh-PP conditions (Estimate = –0.006, SE = 0.01, *t* = –0.71, *p* = 0.479).

## Discussion and conclusion

The present study conducted a large-scale replication of Yoshida et al. (2013) using a lexicality maze task to explore the predictive structure-building process. The experiment revealed gender mismatch effects at the reflexive, which crucially did not interact with wh-type. The absence of the wh-type by gender interaction suggests that, at the wh-phrase, the parser constructs the entire wh-clause with its subject NP coindexed with the first clause subject NP, irrespective of the type of wh-phrase. This finding is partially inconsistent with Yoshida et al., who observed gender mismatch effects only in the wh-NP conditions. Additionally, analysis revealed garden-path effects at the disambiguating region in both wh-NP and wh-PP conditions, supporting the evidence that the parser predictively constructs the wh-clause in these conditions. The presence of garden-path effects also indicates that the parser attempts to construct the globally grammatical structure upon disambiguation, a finding against one possible reflex of Good-Enough language comprehension. Analysis also revealed that garden-path effects are present at the post-disambiguating region only in the wh-NP condition. This observation suggests two loci of garden-path effects in this condition (i.e., the wh-clause subject NP and verb).

The finding that the parser predictively constructs the clausal structure in both wh-NP and wh-PP conditions suggests that the predictive mechanism of sentence parsing is more powerful than assumed by Yoshida et al. (i.e., parallelism-driven prediction). What mechanism underlies the predictive parsing process?

Language comprehension theories often assume left-corner parsing (a class of top-down parsing algorithms; see Aho and Ullman 1972; Grune and Jacobs 2008; Johnson-Laird 1983) as the underlying mechanism of sentence parsing. However, this algorithm is not powerful enough to project the entire clausal structure at the wh-phrase (see Yoshida et al. 2013, pp. 290–291). As noted in the Introduction, prediction in both wh-NP and wh-PP conditions is explicable if we assume that the human parser constructs obligatory structures immediately and incrementally (recall that the clausal structure after the wh-phrase is necessary for the sentence’s well-formedness). The incremental licensing theory views online sentence processing as a process of immediate incremental satisfaction of grammatical constraints (e.g., Abney 1986; Aoshima et al. 2004; Crocker 1996; Frazier and Clifton 1996; Gibson 1991; Gibson et al. 1994; Gorrell 1995; Pritchett 1988, 1991, 1992; Weinberg 1999). In the wh-NP and wh-PP conditions, the appearance of the wh-phrase indicates the beginning of a TP, which must contain a subject NP, a VP and the wh-phrase’s base position. Therefore, the incremental licensing parser should construct the entire wh-clause upon encountering the wh-phrase, which explains the prediction process observed in the present study.

Another issue to discuss in relation to the predictive mechanism is why the parser posits coreference between the subject NPs of the first clause and the wh-clause. As noted in the Introduction, Yoshida et al. (2013) argue that the parser maximises parallelism between the two clauses, leading to joint reference in the wh-NP conditions. In the wh-PP conditions, parallelism does not hold. However, the two clauses still share some similarities in syntactic structure and lexical content. For example, the incremental licensing parser recognises that these clauses consist of similar, though not identical, syntactic structures and share a lexical item (e.g., “stories”) that follows each other’s VP (e.g., [_CP_ [_TP_ [_NP_ Janet’s grandfather] [_VP_ told [_NP_ some stories]]]] … [_CP_ [_TP_ [_NP_] [_VP_ [_PP_ with which stories]]]]). Also, the wh-phrase (“which stories”) referentially relates to the NP (“some stories”) in the first clause (assuming footnote 1). These similarities may lead the parser to conceive of the two clauses as related and expect them to be as homogeneous as possible, resulting in joint reference between the two subject NPs ([_TP_ [_NPi_] [_VP_ V1]] … [_CP_ [_PPt_ wh] [_TP_ [_NPi_] [_VP_ V2 t]]], V1 ≠ V2). This hypothesis explains why the parser does not assume coreference with the possessive NP. Recall that the present study tested the experimental materials used in Yoshida et al. In these materials, the first clause subject NP had a possessive NP that always differed from it in gender (e.g., “Janet/Justin’s grandfather/grandmother”). The results suggested that this gender manipulation did not affect reading times at the reflexive in the wh-PP conditions. We can explain this observation by assuming that the parser expects the maximised similarity between the two clauses in the case of the prepositional wh-phrase.

Alternatively, the parser may favour joint reference because integrating a new referent into the current structure incurs processing costs. Some research in the literature has proposed analogous concepts (e.g., Altmann and Steedman 1988; Gibson 1998, 2000). Gibson (1998), for example, argues that intervening elements that introduce a new referent increase memory costs, resulting in processing difficulty. In the wh-NP and wh-PP conditions, the wh-clause subject NP intervenes between the landing site and the base position of the wh-phrase. Therefore, Gibson’s hypothesis predicts increased memory costs when the embedded subject NP introduces a new referent. Given that the parser often disfavours costly analyses during sentence processing (e.g., De Vincenzi 1991; Fodor and Inoue 2000; Frazier 1979), the postulation of joint reference between the subject NPs may be (partly) due to the avoidance of a new referent, especially in the wh-PP conditions.^4^

**Fig. 3:**
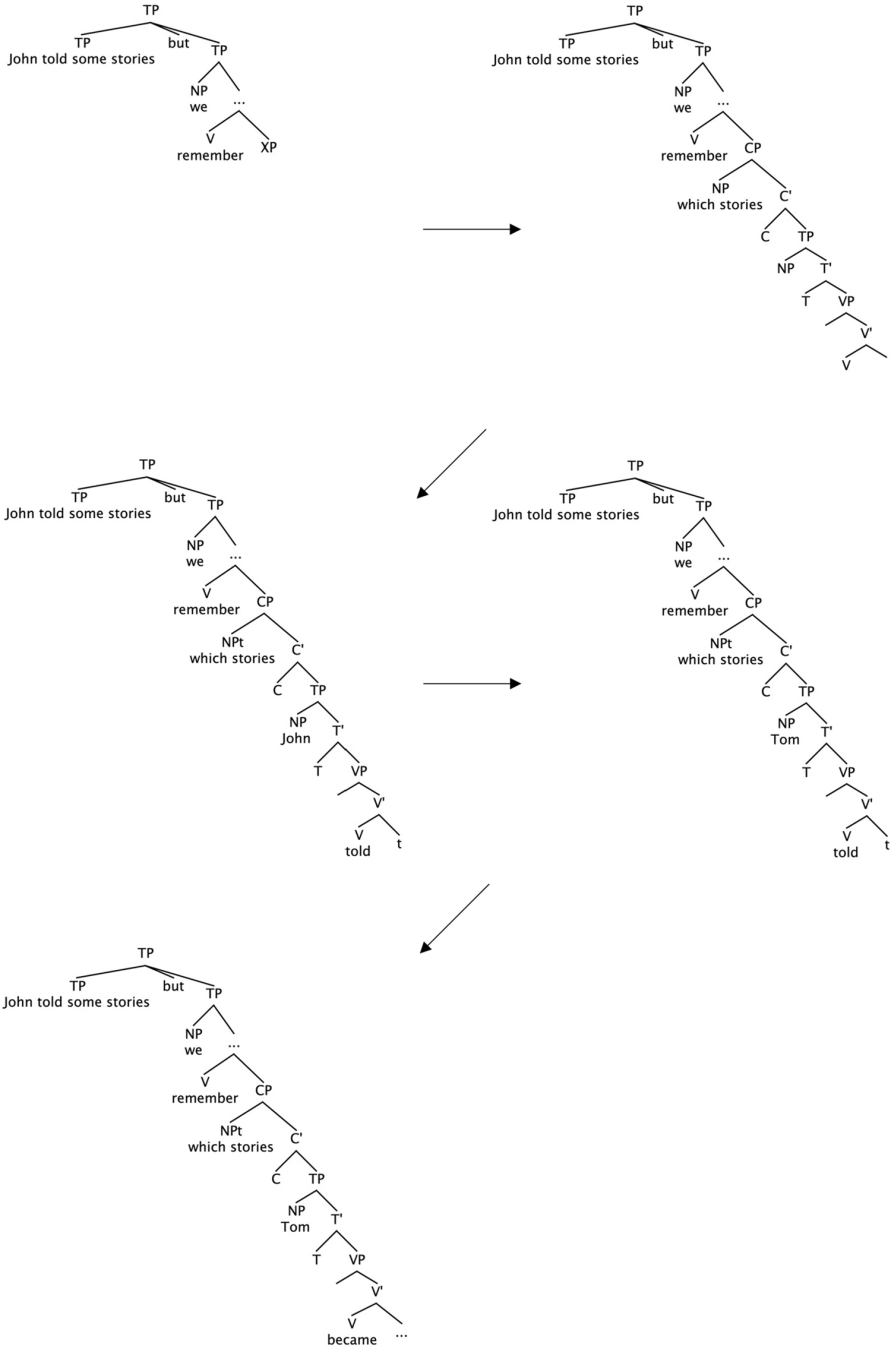
A parsing process in the wh-NP condition (John told some stories but we couldn’t remember which stories Tom became impressed with)

**Fig. 4:**
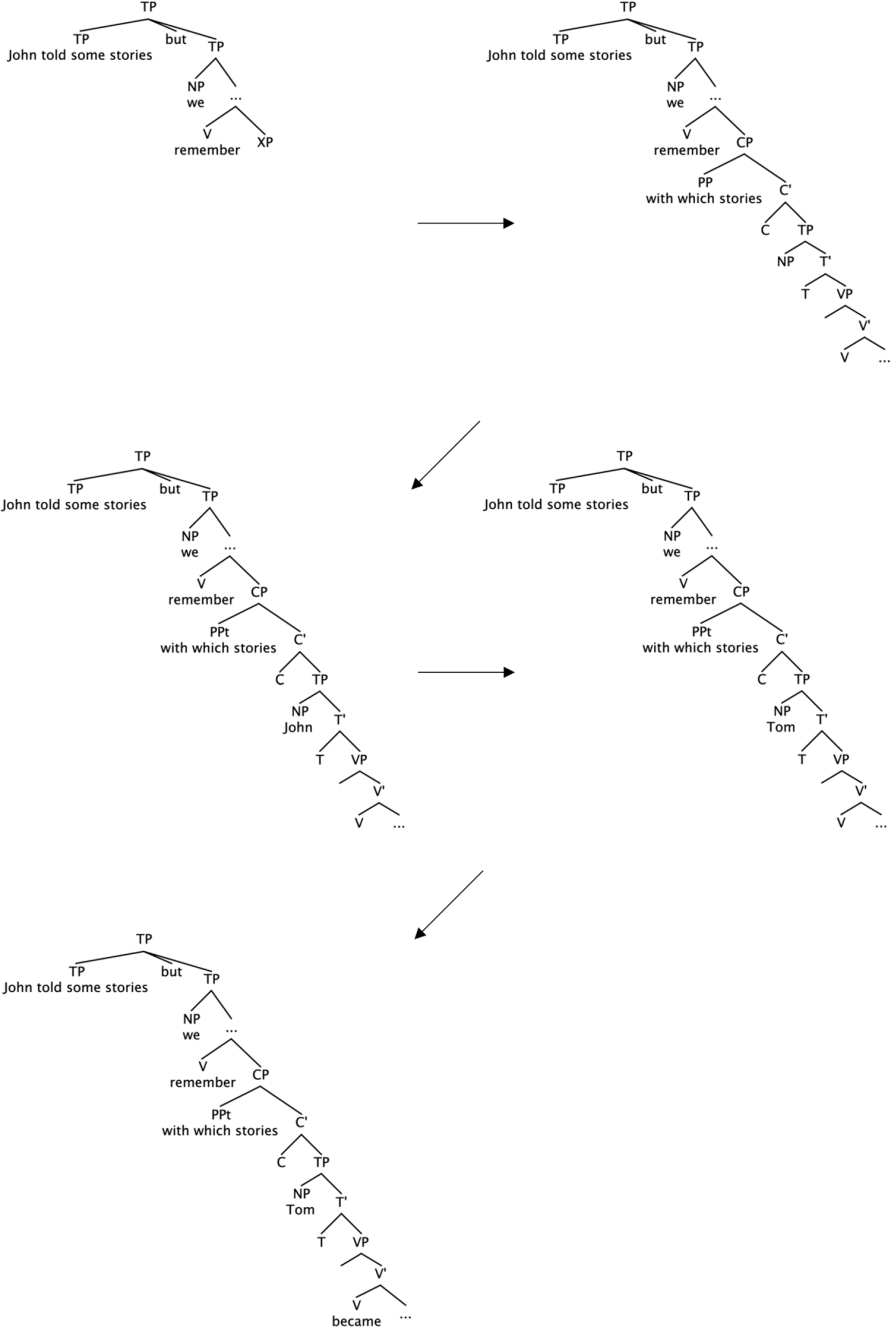
A parsing process in the wh-PP condition (John told some stories but we couldn’t remember with which stories Tom became impressed)

To summarise the discussion thus far, the parser predictively constructs the entire wh-clause upon encountering the wh-phrase to satisfy grammatical constraints immediately and incrementally. The parser then expects the conjoined strings to be maximally similar and/or attempts to avoid a new referent, leading to joint reference between the two subject NPs. Thus, the parser recovers the wh-clause’s entire content (the subject NP and VP) from the first clause in the wh-NP conditions, whereas, in the wh-PP conditions, it recycles only the subject NP. This hypothesis is compatible with what we observed at the post-disambiguating region (i.e., the wh-clause verb). Recall that in the wh-NP conditions only, the post-disambiguating region showed garden-path effects. This finding is explicable if we assume that the parser needs to revise the wh-clause subject NP and VP in the wh-NP conditions but only the wh-clause subject NP in the wh-PP conditions (see Figs. 3 and 4).

Lastly, I discuss the possibility that different observations between Yoshida et al. (2013) and the present study are due to differences in the tasks employed. As mentioned in the Introduction, Yoshida et al. measured reading times using self-paced reading, whereas the present study utilised a lexicality maze task. One unique feature of maze tasks that may have influenced the results is that they compel an incremental analysis of each word due to the forced choice between two candidates (e.g., Forster et al. 2009), which may prevent readers from strategically creating underspecified representations while they read. In other words, data from self-paced reading tasks may often reflect strategic underspecification rather than the intrinsic nature of the human parser when they indicate processing patterns incompatible with the principles of grammar (which can sometimes lead to the misapprehension that underspecification is a general property of sentence processing). Note that I do not intend to argue that maze tasks are superior to self-paced reading tasks. My point is that self-paced reading may be a more advantageous tool for investigating behaviouristic aspects of language comprehension (e.g., underspecification) as observed in a natural reading setting, whereas maze tasks may be a more appropriate choice for inquiry into the mechanism underlying sentence processing. Under this hypothesis, it is conceivable that different observations between Yoshida et al. and the present study, such as the presence or absence of garden-path effects, result from task-specific strategies.

In conclusion, the present study suggests/corroborates the following:

1. The human parser predictively constructs hierarchical syntactic structures during online sentence processing.
2. This predictive structure building results from the parser’s attempt to satisfy grammatical constraints at the earliest opportunity.
3. The parser attempts to maximise the similarity between the conjoined strings, which results in the recovery of the entire or partial content of the wh-clause from the first clause.
4. The parser may also posit joint reference between the subject NPs of the clauses to avoid processing costs incurred by integrating a new referent into the current structure.
5. When disambiguating input indicates that the predictively built structure is globally ungrammatical, the parser conducts revision to construct the globally grammatical structure.

## Data Availability

Data and analysis code are available at https://osf.io/rh4xz.

## Footnotes

1 In the sentences in question, such as (1/2), I assume that the parser analyses the NP headed by a noun in the wh-phrase as coextensive with the one in the first clause.

2 Yoshida et al. (2013) based their study on a linguistic phenomenon called *sluicing* (e.g., Chung et al. 1995; Frazier and Clifton 1998; Merchant 2001; Ross 1969; Yoshida et al. 2014), which is a subclass of *ellipsis* that refers to the omission of linguistic expressions (e.g., Fiengo and May 1994; Lobeck 1995; Phillips and Parker 2014; Ross 1969; Sag 1976; Williams 1977). In (1), for example, the wh-clause’s content recovered from the first clause can be elliptical. The crux of the present study is whether the parser predictively constructs the clausal structure, recognises parallelism and reuses material from the left context, and whether the parser favours ellipsis is of little importance for the present study. Therefore, I do not discuss issues about ellipsis.

3 The cut-off thresholds used in the present study are longer than those adopted in many language comprehension studies. I analysed the data using different cut-offs (between 200 and 300 ms and between 5000 ms and 10000 ms), but all analyses showed similar results.

4 This hypothesis may also explain part of the online *cataphora* resolution process (e.g., Ackerman et al. 2015; Giskes and Kush 2021; Kazanina et al. 2007; Kush and Dillon 2021; van Gompel and Liversedge 2003). Consider the following sentence as an example of a cataphor. (5) Before he left, John had lunch. The sentence in (5) contains a pronoun. The parser can resolve this pronoun by either searching for its antecedent in the right context (cataphora) or taking an extrasentential antecedent. There is evidence that the parser prefers cataphora (e.g., van Gompel and Liversedge 2003), which may be attributable to its attempt to avoid a new referent.
